# The Gradual Reduction vs. the Hyoscine or Quick Withdrawal Method in the Treatment of the Morphine Habit

**Published:** 1912-03

**Authors:** W. C. Ashworth

**Affiliations:** Greensboro, N. C.; Superintendent Telfair Sanitarium


### THE GRADUAL REDUCTION VS. THE HYOSCINE OR QUICK WITHDRAWAL METHOD IN THE TREATMENT OF THE MORPHINE HABIT.

By W. C. Ashworth, M. D., *Greensboro*, N. C.
*Superintendent Telfair Sanitarium.*

The history of opium using in some form dates back almost from time immemorial. The original home of the poppy was in the Valley of the Nile, and the use of the drug antedates by several centuries the earliest Egyptian civilization. In the Odyssy Homer mentions a mysterious drug that was sent to Helen of Troy, and we have reason to suppose that he refers to opium. It is said that Hippocrates, the Father of Medicine, recommended opium for his patients, and we are told that the ancient priests indulged in its use, being very enthusiastic as to its value to the world. They believed that the opium led the mind into the realms of the Spirits and that the sleep following its use was a bridge to connect the mortal world with the home of the gods.

There are many curious legends concerning the discovery of the effects of opium. One of them has it that an Egyptian prince was lost on a hunting tour and, becoming exhausted, he was carried to a hut and placed on a bed of poppy leaves. There he slept for two days, finally arousing with unusual vigor, mentally and bodily. This story seems to have been credited and to have formed the basis of many others concerning the medical value of poppy leaves, which appealed to the people as being a remedy direct from the hands of the Deity.

Morphinism is, however, a modern disease, that threatens to be one of the most serious menaces accompanying twentieth century civilization. Neurasthenia and brain storms are new differentiations of nervous defects incident to the times, and morphia is the new solace which gives temporary relief, while masking the real condition and increasing and intensifying the trouble.

But close on the realization of the danger that lurks in the veins of the poppy came the demand for remedial measures in the cases of those who had succumbed to the seductive poison; and morphinism and its treatment is a subject that calls for the conscientious consideration of every physician who handles the drug to-day.

The literature on the treatment of drug addictions is so meagre that I will be compelled to speak largely from the experience of my calling, which has caused me to be almost constantly associated with this class of patients for a number of years. I have sat at the same table with them; I have watched at their bedside, day and night; I have taken part in their recreations; I have listened to their histories and received their confidences, and studied their characters asleep and awake. The result of several years of close, daily association with these men and women is an overwhelming amount of evidence in favor of the theory that morphinism is a disease rather than a voluntary vice.

The invention of the hypodermic needle by Pravoz, in 1865, and the introduction of its use by physicians in the French army, inaugurated a new era in dermatic medication.

One of the first and most serious phases of morphinism with which we have to contend is the profound fascination of the needle. In most cases the rapidity and certainty of its effects without unpleasant consequences leaves a mental impression not easily effaced. The restful calm which follows the prick of the needle is both psychological and physiological in its effects. It is now recognized that there is a veritable needle mania, demanding that drugs be taken in this way. There is a confidence on the part of the users that the power of the drug will be increased and the action more certain if taken by means of the needle.

Some cases of needle mania are very interesting. I have had patients beg me for the needle prick, knowing at the same time that no effect other than psychic would be produced. I have seen these patients prick themselves with rusty needles that had been discarded as worthless.

The craving for morphine is more persistent and difficult to overcome than the desire for whiskey, and the effects of the drug are far worse upon the psychical and mental organization. The higher the brain culture and development, the more certain and persistent are the disastrous effects of morphine. The early use of morphine, and sometimes its continued use for many years, may exhibit but little physical impairment in the habitue, but in all cases the will and moral forces suffer from the beginning.

Morphinism is classed by some authorities as a form of insanity, and certainly the use of the drug gradually leads to diseases of both the brain and nervous system. The constant narcotism of the higher brain centers impairs their integrity and destroys their normal activity. My observation is that when the craving for morphine becomes serious and imperative during the withdrawal of the drug, the indications are unmistakable of serious and possible permanent impairment.

The fascinating physiological action of morphine, which enable the nervous and confused brain worker to regain his mental clearness and self-possession after taking a single dose of this drug, creates a demand for its use that threatens the highest moral and mental forces of the times. One dose will enable the overworked, wornout physician to take up the labors of his arduous profession with new energy and enthusiasm for the time being, but alas! the effects of the one dose do not suffice for long, and it must be repeated again and again until the man, who thought to simply be tided over a crisis in his work, finds himself in the toils of the enslaving tyrant.

The extent of drug using is appalling and far beyond the imagination of the general practitioner. There are many causes assigned for the increase in the use of opiates, and all, no doubt, contribute their quota to the sum total of results.

That the state-wide prohibition law recently enacted, but poorly enforced in most localities, is contributing much to the inordinate use of drugs, can not be denied. It is apparent to the most casual observer that the whiskey now used by most whiskey drinkers is of very inferior quality, and as a consequence the injurious effects are more pronounced. The nervous system of the consumer of the cheap chemical whiskey is soon wrecked; therefore the transition to the use of drugs is easy and rapid. I think I am safe in saying that fully eighty per cent. of all drug users give a frank confession of having used whiskey to excess. The unfortunate result is natural and the reason obvious when we take into consideration the fact that there must come a time in the history of every man who drinks whiskey to excess when the whiskey fails to satisfy the abnormal craving for artificial stimulation.

Just here I wish to utter a word of warning to physicians against the common practice of administering morphine to their inebriate patients. The nervous system of the intoxicated patient is abnormally sensitive to morphine, and it is a very easy matter to supplant the whiskey habit by producing a case of morphinism.

Without doubt another important factor contributing in no small degree to this widespread disease, is the strenuous life necessary to meet the increased cost of living. It seems that almost numberless people must resort to opiates and stimulants to keep up their flagging courage and energy.

Authorities differ very widely as to the best course to pursue in the treatment of the drug habit, but it is generally conceded that the treatment should be essentially eliminative in its nature rather than sedative. Opiates which check the secretions should be avoided as much as possiblt. One of the first requisites in the successful treatment of morphinism is to recognize that it is a disease. Unnecessary as this statement may seem, we find many who do not believe it to-day. It stands to reason that the doctor who starts with the belief that his patient has no disease is not likely to effect a cure.

To find a substitute drug which will completely and safely relieve the discomfort and uneasiness following the withdrawal of morphine is the dream of the credulous physician and the boast of the charlatan and quack, but so far it is unrealized. The action of hyoscine on a highly sensitive, irritant brain may produce disturbances and mental conditions of great danger. Like chloral, the pleasing narcotism and sleep which follows its use may develop congestions and irritations of a most serious nature. It is certainly a very toxic drug, and its peculiar action should be recognized in all confused cases.

My experience with hyoscine leads me to believe that that method of treatment is rather noted for its quick cures and correspondingly quick relapses. The weakened nerve centers call for prolonged tonic treatment rather than the sudden shock incident to the sudden withdrawal or hyoscine method.

The temporary cure of the morphine habit is usually a simple matter if the patient is confined and hyoscine is administered, but the permanent cure of the habit by this method is a very rare occurrence, as those experienced in this line of work will admit. Statistics on the result of the treatment of the morphine habit are useless unless they show permanency of results, and I have rarely seen those who did when the hyoscine method was employed.

Another objection to the hyoscine method is the fact that nearly all morphine users are borderland patients mentally; consequently the use of hyoscine is contraindicated from this fact alone. The reconstruction of the patient's will power and self-control, which is the sine qua non to a permanent cure, can not be accomplished by the hyoscine method. The best possible opportunity for such reconstruction is offered by the GRADUAL WITHDRAWAL METHOD. We are not re-educating self-control when we force oblivion on the patient by the use of hyoscine.

I use the Gradual Reduction method of treatment, coincident with the administration of strong reconstructive tonics, which enables me in most instances to withdraw the morphine with only a negligible amount of discomfort. In fact, the discomfort is so slight that the patient is seldom aware when the drug is entirely withdrawn. The reduction is made in a very tentative manner, and never so rapidly as to produce any undue shock to the nervous system or circulation.

The rapidity of the reduction should be regulated by the tolerance of the individual as manifested by the withdrawal symptoms. During the period of reduction the patient should be safeguarded but not incarcerated. I do not believe in treating the average morphine patient as an irresponsible, but rather in securing his confidence by giving him mine and the assurance that I will do all in my power to help him make the fight and prevent any serious suffering.

The Gradual Reduction method offers the greatest advantages for the building up of the broken down nervous system and the re-education of the deficient will power and self-control. It requires time to drill into the narcotic mind of the patient that it is possible to live without artificial stimulation. Extensive experience in the treatment of the morphine habit and close study of the experience of others convince me that the patient should be backed out of the slough of his habit in very much the same manner that he entered, namely; rapidly or moderately, according to the way in which he became addicted to the use of the drug. The immediate withdrawal of the morphine shocks the nervous system so severely as to permanently weaken it, and consequently, the subsequent self-control of the patient. For this reason I advise the withdrawal of the drug along the same general reverse lines as the periodical increasing dosage of the drug was acquired.

No individual can be permanently cured of the morphine habit without active, scientific help. I believe that the dosage should be lowered to a certain point and held there for several days without attempting further reduction until the patient is accustomed to the lessened amount. In this way, the withdrawal symptoms are usually limited to the first few days of reduction. I always advise a sharp reduction to start with, as most patients are taking a great deal more morphine than they need to prevent unbearable symptoms.

In the average case the total withdrawal should not be undertaken until the patient is down to a very small daily amount, say about 1-16 of a grain in twenty-four hours. I always give the last dose at bed-time when the patient is only taking one dose daily.

The morphine should be given hypodermatically, regardless of the former method of taking it by the patient, and combined with each dose should be given strychnine, sparteine and such other stimulants as will best support the circulation.

To promote sleep during the time just prior to, and following the withdrawal of the drug, I use bromide of sodium, hot spinal fomentations, electric light baths, veronal sodium, trional, etc., but never use chloral. For the intercurrent nervous symptoms and the pain incident to the withdrawal period, aspirin, gelsemine vibratory massage, hot baths, etc., have proven of marked benefit.

Give an abundance of purgatives throughout the treatment and less morphine will be required. Many of the withdrawal symptoms are the result of auto-intoxication produced by the enervated condition of the bowels and stomach as a result of the morphia.

The diet should be bland and unirritating, especially during the final withdrawal period. Often the craving is greatly increased by acute indigestion produced by overeating. A diet should be outlined that is adequately balanced and absolutely free from food leaving much residue or that is apt to cause fermentation.

Bothm Erlenmeyer and Jennings are strong advocates of the use of bicarbonate of soda for the relief of indigestion during the treatment. In fact Erlenmeyer terms it "Chemical demorphinization."

The symptoms following the withdrawal of the drug are real symptoms, although the patient may exaggerate them. But the physician who fails to realize the positive reality and severity of the symptoms following the withdrawal of the morphine will usually fail in the permanent cure of the habit. It is not difficult for him to diffeentiate between the assumed and the real, because there are so many objective symptoms that may be easily detected.

The central fact to be remembered is that the withdrawal of the morphine is not the end, but only the beginning of the treatment. A long period of building up and restoration must follow before the brain and nervous system can attain the degree of vigor essential to control the neurotic symptoms which appear in the wake of the morphine.

During the reconstruction period the patient should be regarded as a nervous bankrupt. All physical or mental strain should be avoided and cheerful surroundings with light mental diversion are necessary adjuncts to the reguar treatment. The patient should not be allowed to return to his customary occupation for several weeks after the withdrawal of the drug.

I have given only a brief resume of the treatment to be used in the relief of morphinism as it must in all cases be modified to suit the temperament and idiosyncrasies of the patient. Before accepting a patient the physician should have it plainly understood that he is to have absolute control of the patient throughout the treatment, also that he does not intend to incarcerate the patient, but that certain restrictions will be imposed which, if disregarded, will relieve the doctor of all responsibilities in the case.

There is no royal road to the cure of the morphine habit, and there will never be any treatment which does not entail a certain amount of discomfort on the part of the patient. When you hear of a case of morphinism cured without any suffering, you may be assured that the patient was surreptitiously taking the drug during the "cure" and is most likely taking it now.

This is an old subject in many respects and, threatening as it does the very flower of culture and morality, it is one that cries for our earnest attention. The successful annihilation of this dread monster is a difficult question to solve.

What then? shall we sit idly down and say
The night hath come; it is no longer day?
The night hath not yet come; we are not
Quite put off from labor by the failing light;
Something remains for us to do or dare;
Even the oldest tree some fruit may bear.

